# Low Secretory Leukocyte Protease Inhibitor (SLPI)-Level Potentiates Alternaria Extract-Induced T-Helper 2 (Th2) Airway Inflammation via the Interleukin-33 (IL-33) Pathway

**DOI:** 10.7759/cureus.84488

**Published:** 2025-05-20

**Authors:** Taizo Hirano, Akira Koarai, Shinya Ohkouchi, Hisatoshi Sugiura, Hajime Kurosawa

**Affiliations:** 1 Department of Occupational Health, Graduate School of Medical Sciences, Tohoku University Graduate School of Medicine, Sendai, JPN; 2 Department of Respiratory Medicine, Sendai City Hospital, Sendai, JPN; 3 Department of Respiratory Medicine, Graduate School of Medical Sciences, Tohoku University Graduate School of Medicine, Sendai, JPN

**Keywords:** asthma, il-33, secretory leukocyte protease inhibitor (slpi), serine protease, type 2 innate lymphoid cells (ilc2s)

## Abstract

Introduction

Serine proteases play a critical role in the augmented release and cleavage of IL-33, leading to the expansion of group 2 innate lymphoid cells (ILC2s) and T-helper 2 (Th2) airway inflammation. However, the protective regulation of protease-dependent interleukin-33 (IL-33) activation remains poorly understood. Therefore, we investigated the role of secretory leukocyte protease inhibitor (SLPI), as a serine protease inhibitor, in this protective regulation and aimed to clarify its contribution to type 2 immunity.

Methods

We evaluated the role of SLPI in the *Alternaria* extract-induced expansion of ILC2s and Th2-type airway inflammation via IL-33, using three models: SLPI-deficient mice, an in vivo SLPI knockdown model with shRNA, and an in vitro model utilizing primary human bronchial epithelial cells (HBECs) exposed to *Alternaria* extract under various conditions, including plasmid transfection.

Results

We showed that two mouse models of downregulation of* SLPI *gene expression augmented *Alternaria* extract-induced release of IL-33 and the expansion of ILC2s, together with Th2 airway inflammation. Furthermore, two treatment models using *SLPI* KO mice, administration of a serine protease inhibitor, bovine pancreatic trypsin inhibitor, or anti-IL-33 antibody, attenuated Th2 airway inflammation. In two in vitro experiments, SLPI, as a serine protease inhibitor, prevented both the release of IL-33 from HBECs and the cleavage of full-length IL-33 to shorter mature forms by neutrophil elastase.

Discussion

These findings suggest that SLPI functions as a key serine protease inhibitor in regulating IL-33-mediated type 2 immune responses. Low SLPI levels may contribute to the pathogenesis of asthma by promoting Th2 inflammation, highlighting SLPI as a potential therapeutic target in patients with low SLPI expression.

## Introduction

Asthma comprises two principal immunophenotypes, T2-high asthma, which is predominantly allergic and driven by T-helper-2 (Th2) inflammation, and T2-low asthma, which is largely non-allergic, yet both are clinically characterized by variable expiratory airflow limitation and airway hyperresponsiveness. Because allergen exposure preferentially amplifies the T2-high endotype, whereas respiratory infections often provoke T2-low or mixed responses, controlling the rapid induction of Th2-type inflammation remains a critical objective in current asthma treatment strategies [1].

Interleukin-33 (IL-33), a cytokine belonging to the interleukin-1 (IL-1) family, plays a key role in tissue homeostasis, repair, and type 2 immunity. When cellular damage or inflammation occurs after allergen exposure or infections, IL-33 is released from the damaged cells and activates various immune cells, including mast cells, type 2 helper T lymphocytes, and group 2 innate lymphoid cells (ILC2s) [2]. ILC2s, which are major targets of IL-33 and produce large amounts of Th2 cytokines such as interleukin-5 (IL-5) and interleukin-13 (IL-13), both of which play critical roles in the rapid induction of type 2 immunity [3].

Recent genome-wide studies have identified a major asthma susceptibility locus near the IL-33 gene, correlating with disease severity [4]. In a mouse model, the deficiency of IL-33 has been shown to decrease allergen- or protease-induced type 2 airway inflammation [5]. These findings implicate IL-33 in the pathogenesis of asthma. Recent studies have reported that protease activity possessed by allergens such as *Alternaria alternata *(*Alternata*) increases the expression of IL-33 in airway epithelial cells [6]. Furthermore, proteases not only increase IL-33 but also enhance its activity by cleaving the full-length form of IL-33 (IL-33FL) into shorter mature forms [7]. Collectively, these studies have revealed crucial roles of proteases in the expression and activation of IL-33.

Secretory leukocyte protease inhibitor (SLPI) is an inhibitor of serine proteases, including human neutrophil elastase (NE), and it possesses antimicrobial, antiviral, and anti-inflammatory activities partly through suppression of nuclear factor-κB signaling [8,9]. In asthma mouse models, both *SLPI*-knockdown and overexpression experiments have demonstrated a protective role of SLPI in allergic airway inflammation [10,11]. In severe asthma patients, decreased SLPI expression in airway epithelial cells suggests that the loss of SLPI’s protective function contributes to disease pathogenesis [12]. Furthermore, an in vitro study revealed that SLPI inhibits ATP-induced IL-33 production in type II alveolar epithelial cells [13]. From these findings, SLPI may be involved in the pathogenesis of protease-mediated Th2-type inflammation, exemplified by asthma, by modulating the innate immune response, principally through attenuation of IL-33 production.

Against this background, we sought to clarify whether reduced SLPI indeed unleashes protease-dependent IL-33 maturation and downstream Th2-type inflammation in vivo and in vitro. We therefore investigated the protective role of SLPI in protease-dependent IL-33 expression and activation after *Alternaria* exposure using both genetic downregulation of *SLPI* and an in vitro model with primary human bronchial epithelial cells (HBECs), which were exposed to *Alternaria* extract under various conditions. Here, we found that reduced SLPI levels in airway epithelial cells enhance IL-33 expression and potentiate IL-33FL cleavage into shorter mature forms, resulting in the expansion of ILC2 populations and amplification of Th2-type airway inflammation.

## Materials and methods

All animal procedures were approved by Tohoku University’s Institutional Committee for Laboratory Animal Care.

Mice

C57BL/6 mice were purchased from Japan Charles River (Yokohama, Japan). *SLPI*-deficient (*SLPI* KO) mice were generated as described previously [10]. Briefly, genomic DNA containing the *SLPI* gene was isolated from a 129/Sv mouse genomic library and subcloned into the pBluescript SK(-) vector. A targeting vector replacing all four *SLPI* exons with a phosphoglycerate kinase (PGK)-driven neomycin resistance cassette was electroporated into E14.1 embryonic stem (ES) cells. G418-resistant clones were screened for homologous recombination by Southern blotting. Targeted ES clones were microinjected into C57BL/6 blastocysts to generate chimeric mice, which were then crossed with C57BL/6 females to obtain heterozygous offspring. All animals used in this study were matched for age (6-8 weeks old), sex (female), and strain (C57BL/6 background) and were housed under specific pathogen-free conditions.

*Alternaria* extract-induced airway inflammation model

Mice were intratracheally injected with *Alternaria* extract (50 μg, Greer Laboratories (Lenoir, N.C., USA) in a total volume of 50 μl in phosphate‐buffered saline (PBS) at pH 7.0, once a day for three days. Twenty-four hours after the final injection, bronchoalveolar lavage (BAL) was performed, and lung tissues were collected for histopathological analysis. For the BAL examinations, the lungs were lavaged twice with 0.75 ml of PBS. After centrifugation, total BAL cells resuspended in 1 ml of PBS were counted with a handheld automated cell counter (ScepterTM, Millipore, Bedford, MA). Differential BAL cell counts were determined for macrophages, lymphocytes, neutrophils, and eosinophils on the basis of the cellular characteristics by counting a minimum of 200 total cells after preparation with cytospins and staining with Diff-Quik (Sysmex). For the histopathological examination of lung tissue, paraffin sections (5 μm thick) were fixed with 10% formaldehyde and stained with hematoxylin and eosin (H&E) or periodic acid-Schiff stain (PAS). 

In vivo treatment with BPTI or anti-IL-33 antibody

To investigate the role of serine protease, the serine protease inhibitor BPTI (1mg in 50μl, Sigma, St. Louis, MO) or normal saline was injected nasally into the mice 1 hour after *Alternaria* extract injection for three days. To evaluate the effect of IL-33, anti-mouse IL-33 antibody (10mg in 50μl, R&D Systems, Minneapolis, USA) or control antibody was injected nasally into the mice 1 hour after *Alternaria* extract for three days.

In vivo and in vitro knockdown of the *SLPI* gene

Plasmid vectors expressing short hairpin RNA (shRNA) under the control of the mouse U6 promoter were constructed using pBAsi-mU6 Pur DNA (Takara Bio, Otsu, Japan). The pshSLPI plasmid vectors expressing shRNA under the control of the mouse U6 promoter were constructed using pBAsi-mU6 Pur DNA (Takara Bio, Otsu, Japan). The pshSLPI vector and the pshCtrl vector express shRNA against the *SLPI* gene and the control scrambled gene, respectively. The shRNA target sequences were as follows: pshSlpi, 5’- GCAAGATGTATGATGCTTA-3’, starting at position 518 in NM_011414 of GenBank; pshCTRL, 5’-TACGTATGGATGGATTCAA-3’. Human plasmid vectors expressing shRNA under the control of the mouse U6 promoter within the lentivirus plasmid vector (pLKO.1-puro) were purchased (Sigma-Aldrich, St. Louis, USA, SLPI:NM_003064, Control:SHC002). The plasmids, including pm *SLPI* and pNull, are pBluescript II KS(+) vectors (Agilent Technologies, Santa Clara, CA) in which the mouse *SLPI* cDNA and no transgene, respectively, are under transcriptional control of the cytomegalovirus (CMV) immediate‐early enhancer and promoter. To modify the nucleic acid sequence of pmCMV SLPI, a QuikChange II Site-Directed Mutagenesis Kit (Agilent Technology) was used according to the manufacturer's instructions. For in vivo delivery of these shRNA vectors, small complexes with a linear polyethylenimine (PEI) were prepared using in vivo-jetPEI (Polyplus-transfection SA, Illkirch, France) per the manufacturer’s instructions. The in vivo-jetPEI complexes, including 50 μg of the plasmid vector, were injected nasally into the mice in a total volume of 50 μl of 5% glucose [14].

RNA assessments

Total cellular RNA was extracted from BAL cells by a RNeasy Plus kit (QIAGEN, Valencia, CA). For reverse transcriptase-polymerase chain reaction, total cellular RNA was reverse‐transcribed to cDNA using a high-capacity RNA‐to‐cDNA kit (Life Technologies, Grand Island, NY). Quantitative PCR was performed by a SYBR GreenER qPCR SuperMix Universal kit (Life Technologies) in the DNA Engine Opticon 2 system (Bio‐Rad Laboratories, Hercules, CA) as suggested by the manufacturer. The quantitative data were normalized to the mouse glyceraldehydes‐3‐phosphate dehydrogenase (mouse Gapdh) expression unless otherwise noted, and the relative gene expression was determined as a factor by which the normalized expression of the sample was changed from that of the reference [15]. The primer pairs used in this study were as follows: mouse SLPI, 5′‐GCTGTGAGGGTATAGTGGGAAA‐3′ and 5′‐CGCCAATGTCAGGGATCAG‐3′; mouse Gapdh, 5′‐TGTGTCCGTCGTGGATCTGA‐3′ and 5′‐CCTGCTTCACCACCTTCTTGA‐3′. 

Flow cytometry

Isolated cells were resuspended as whole lung cells in 100 μl of Hank's balanced salt solution (Life Technologies) supplemented with 10 mM HEPES (4‐2‐hydroxyethyl‐1‐piperazineethanesulfonic acid, Life Technologies) and 2% fetal bovine serum (Nichirei, Tokyo, Japan) and were incubated with the following fluorescence‐conjugated monoclonal antibodies for 60 min at 4°C in the dark: FITC anti-CD3ɛ (clone 145-2C11, 1:100, BioLegend, San Diego, CA), anti-CD4 (clone GK1.5, 1:400, BioLegend), anti-CD8α (clone 53-6.7, 1:100, BioLegend), anti-CD11c (clone N418, 1:400, c), anti-FceRIα (clone MAR-1, 1:400, BioLegend), anti-NK1.1 (clone PK136, 1:400, Biolegend), anti-CD19 (clone 6D5, 1:100, BioLegend), anti-TER119 (clone TER119, 1:100, BioLegend), anti-CD5 (clone 53-7.3, 1:100, BioLegend), anti-F4/80 (clone BM8, 1:400, BioLegend), anti-Gr-1 (clone RB6-8C5, BioLegend), phycoerythrin (PE)‐cyanine 7 (Cy7) anti-ST2 (clone DIH9, 1:400, BioLegend), and APC CY7 anti-CD45 (clone 30-F11, 1:400, BioLegend). Stained cells were analyzed and sorted using a FACS Aria II cell sorter and FACSDiva software (BD Biosciences). A viability dye, 7‐amino‐actinomycin D (7‐AAD, Beckman Coulter, Brea, CA), was used to eliminate dead cells. 

Preparation of epithelial cells and the culture conditions

Primary HBECs acquired from lung lobes resected from patients at surgery were cultured as previously described [16]. To investigate the effect of *Alternaria* extract on IL-33 production, HBECs were exposed to 100 μg of *Alternaria* extract in 1 mL of PBS. For plasmid transfection, HBECs were transfected with 5 μg of the plasmid DNA by Lipofectamine Plus reagent (Life Technologies) following the manufacturer's instructions. To investigate the role of protease-activated receptor 2 (PAR2), the cells were exposed to 100 μg of *Alternaria* extract treated with or without ENBD-1068 (10 mM, Enzo Life Sciences, Exeter, UK).To investigate the cleavage assay of IL-33 by neutrophil elastase (NE), the cells were exposed to 100 μg of *Alternaria* extract and NE (0.3 U) with or without recombinant SLPI (100 μg).

Western blotting 

The accumulation of protein from the culture supernatant was carried out using centrifugal filter tubes (Millipore, Amicon Ultracell). An aliquot of culture supernatant (2.5 μg) was separated in a 12% Bis‐Tris gel (Life Technologies) and transferred onto a polyvinylidene difluoride membrane (PVDF, Life Technologies) using the Trans‐Blot semi‐dry electrophoretic transfer system (Bio‐Rad Laboratories, Hercules, CA). After treatment with PVDF Blocking Reagent (TOYOBO, Osaka, Japan), the membrane was probed with a primary monoclonal antibody against mouse monoclonal anti-IL-33 antibody (1:1000 dilution, Enzo Life Sciences, New York, USA) and a horseradish peroxidase (HRP) conjugated secondary antibody against the primary antibody (1:2000, Santa Cruz Biotechnology). The signals were visualized using ECL Western Blotting Detection Reagents (GE Healthcare, Piscataway, NJ).

Immunohistochemistry

For immunohistochemistry staining of lung tissues, 5‐μm cryosections were dehydrated in 100% ethanol and rehydrated in decreasing concentrations of ethanol in PBS. Antigen retrieval was performed by incubation in water‐diluted Histofine (pH 9.0, Nichirei, Tokyo, Japan) and treatment in an autoclave (15 min, 121°C). After the Protein Block Serum‐Free treatment, rabbit anti-SLPI (1:500 dilution, Santa Cruz Biotechnology) was added and incubated overnight at 4°C. Slides were treated with DAB (3,3'-diaminobenzidine, Nichirei). 

Protein cleavage assay

Recombinant human IL-33 (1μg) was incubated with human neutrophil elastase (0.3 U) with or without recombinant human SLPI (100μg) in 25 μl of assay buffer (pH 8.0, 50 mM Tris, 1.0M NaCl, 0.05% Brig-35) for 30 min to 1 h at 37°C. Cleavage products were analyzed by SDS/PAGE and Western blot.

Enzyme-linked immunosorbent assay (ELISA)

IL-4, IL-5, and IL-33 levels in BAL fluid were measured using ELISA kits (R&D Systems), according to the manufacturer’s instructions.

Statistical analysis

Two data sets were compared using Student's unpaired two‐tailed t‐test. For multiple comparisons, post-hoc analysis was performed by Tukey's test. Normal distribution of the data and the equal variances were assessed by histograms and F‐test statistics. P-values of < 0.05 were considered statistically significant. Error bars in the graphical data represent the mean ± standard error of the mean (SEM). All statistical analyses were performed with GraphPad Prism version 8.0.0 for macOS (GraphPad Software, San Diego, CA, USA).

## Results

Downregulation of *SLPI* gene expression increases *Alternaria* extract-induced Th2 airway inflammation

**Figure 1:**
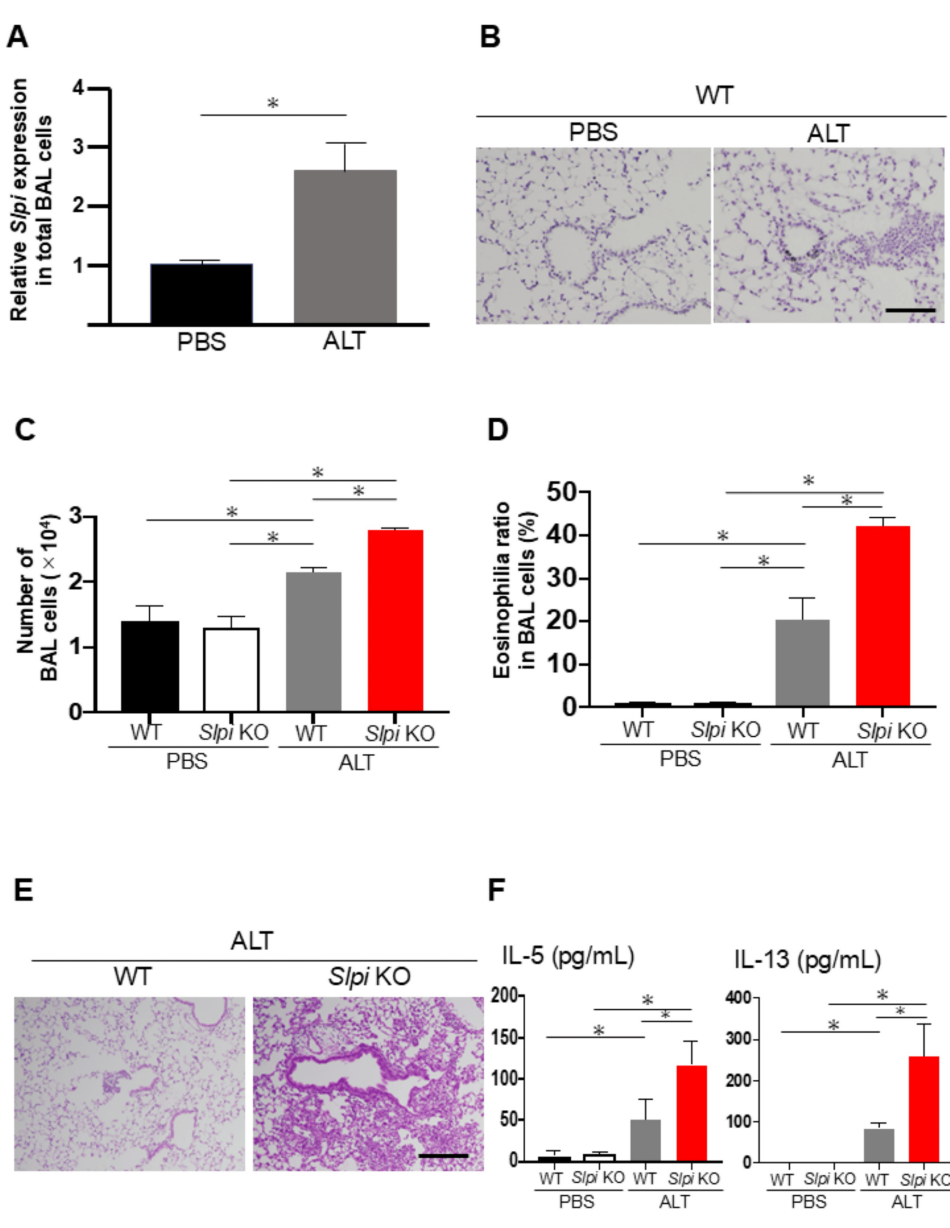
Knockdown of SLPI enhances Alternaria extract-induced Th2 airway inflammation. WT and *SLPI* KO mice were intranasally exposed to *Alternaria* extract or PBS on days 1-3, and analyses were performed on day 4 (n = 3). (A) Relative expression of *SLPI *mRNA in BAL cells was measured by quantitative RT-PCR in WT mice; GAPDH was used as an internal control. (B) Immunohistochemical staining of lung tissue in WT mice showing SLPI protein expression. (C, D) Total BAL cell counts (C) and eosinophil ratios (D) were assessed to evaluate airway inflammation. (E) Representative H&E-stained lung sections (scale bar: 200 µm). (F) Concentrations of IL-5 and IL-13 in BAL fluid. Data are shown as mean ± SEM. Unpaired Student’s t-test (for two groups) and Tukey’s post hoc test (for multiple comparisons); (asterisks indicate p < 0.05).

*Alternaria* extract exposure to the lungs in the early phase is known to elicit innate immunity that provokes Th2-type inflammatory responses. Given the key role of SLPI in the innate immune system, we used two mouse models of downregulated *SLPI* gene, *SLPI* KO mice and an in vivo *SLPI* knockdown with shRNA. As shown in Figures 1A, 1B, exposure of wild-type (WT) mice to *Alternaria* extract increased *SLPI* mRNA levels in the BAL fluid and also elevated SLPI protein levels in peribronchial epithelial cells. Next, we compared airway inflammation between WT and *SLPI* KO mice. Notably, the analysis of total cell counts in the BAL fluid showed that *SLPI* KO mice had significantly increased cells in all counts compared to WT mice (Figure 1C). Similarly, assessment of the lung histology also revealed that marked peribronchiolar and perivascular cellular infiltration was significantly increased in the lungs from *SLPI* KO mice compared to those from WT mice (Figure 1E). Furthermore, assessment of the ratio of eosinophilia in BAL cells and levels of Th2-type cytokines, including IL-5 and IL-13 in BAL fluid, showed a more marked increase in *SLPI* KO mice than in WT mice (Figures 1D, 1F).

**Figure 2:**
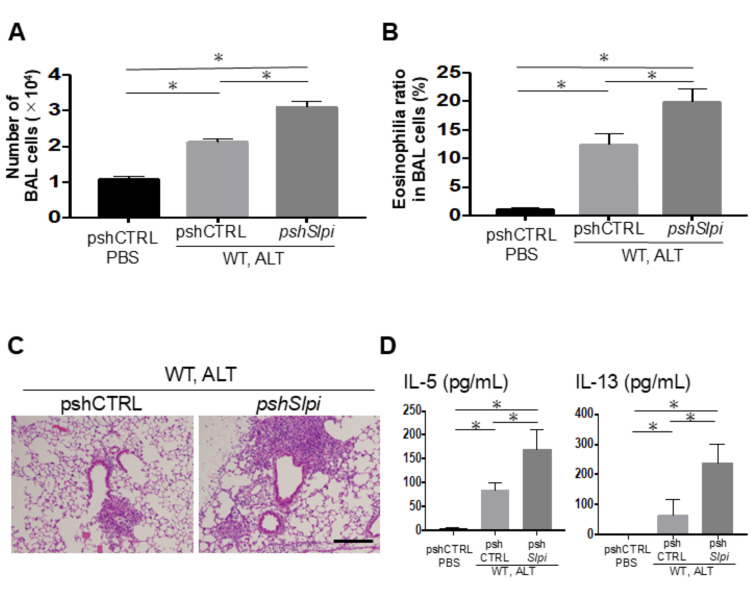
In vivo shRNA knockdown of SLPI enhances Alternaria extract-induced Th2 airway inflammation. These experiments were performed using a protocol similar to Figure 1, except WT mice were intranasally transfected with 50 μg of a plasmid vector expressing either shRNA targeting *SLPI* or a control shRNA (n = 3). (A, B) Airway inflammation was assessed by total BAL cell counts (A) and eosinophil ratios (B). (C) Representative H&E-stained lung sections (scale bar: 200 μm). (D) Concentrations of IL-5 and IL-13 in BAL fluid. Data are shown as mean ± SEM. Unpaired Student’s t-test for two groups; asterisks indicate p < 0.05.

To determine the functional consequences of SLPI in this model, we further analyzed in vivo in the shRNA-mediated knockdown model of the *SLPI* gene. An in vivo *SLPI* knockdown model with shRNA reduced the level of *SLPI* gene expression in lung cells from WT by 64% (not shown). As shown in Figures 2A-2D, a similar trend was observed in two mouse models of downregulated *SLPI* gene. WT mice injected with pshSLPI (*SLPI*-targeting shRNA) also showed enhanced airway inflammation from *Alternaria* extract exposure in the histological examination, total BAL cell count, ratio of eosinophilia in BAL, and levels of Th2-type cytokines (e.g., IL-5 and IL-13) in BAL fluid compared to WT mice injected with control shRNA (pshCTRL).

Downregulation of SLPI expression potentiated the *Alternaria* extract-induced expansion of type 2 innate lymphoid cells via IL-33

**Figure 3:**
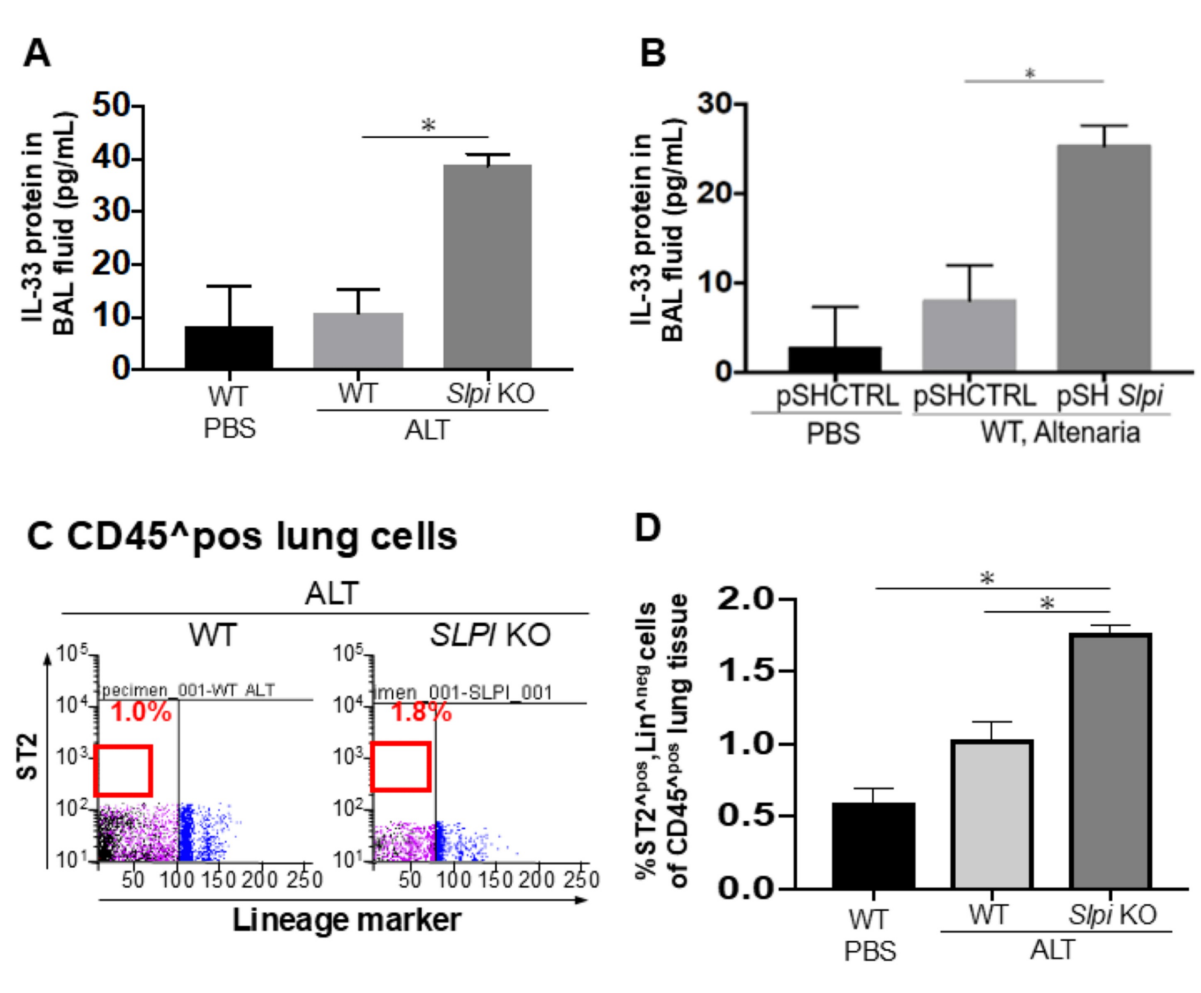
Knockdown of SLPI augments IL-33-dependent expansion of ILC2s. These experiments were performed similarly to Figure 1, except that panel B uses the protocol from Figure.2. (A, B) IL-33 protein levels in BAL fluid were measured by ELISA (n = 3). (C, D) The frequency and number of ILC2s (defined as Lin^neg, CD45^pos, ST2^pos) in lung tissue after exposure to *Alternaria* extract were assessed by flow cytometry. Data are presented as mean ± SEM. Statistical significance was determined by an unpaired, two-tailed Student’s t-test (asterisks indicate p < 0.05).

We hypothesized that IL-33 plays a key role in this augmented type 2 airway inflammation caused by the downregulation of *SLPI* expression. To test this hypothesis, we evaluated the level of IL-33 in the BAL fluid. ELISA revealed that BAL IL-33 levels were significantly higher in *SLPI* KO mice than in WT mice (Figure 3A), and a similar trend was observed in WT mice treated with pshSLPI compared to those treated with pshCtrl (Figure 3B). We next undertook a flow cytometry approach to evaluate the expansion of ILC2s in the lungs after *Alternaria* extract exposure as previously described. The flow cytometry data revealed that the frequency and number of Lin^neg (CD3ε, CD4, CD8α, CD11b, CD11c, CD19, B220, FcεR1α, Gr1, TER-119, and TCRγδ), CD45^pos, and ST2^pos cells, defined as a population of ILC2s, from the lung after *Alternaria* extract exposure were more markedly increased in *SLPI* KO mice than those in WT mice (Figures 3C, 3D).

**Figure 4:**
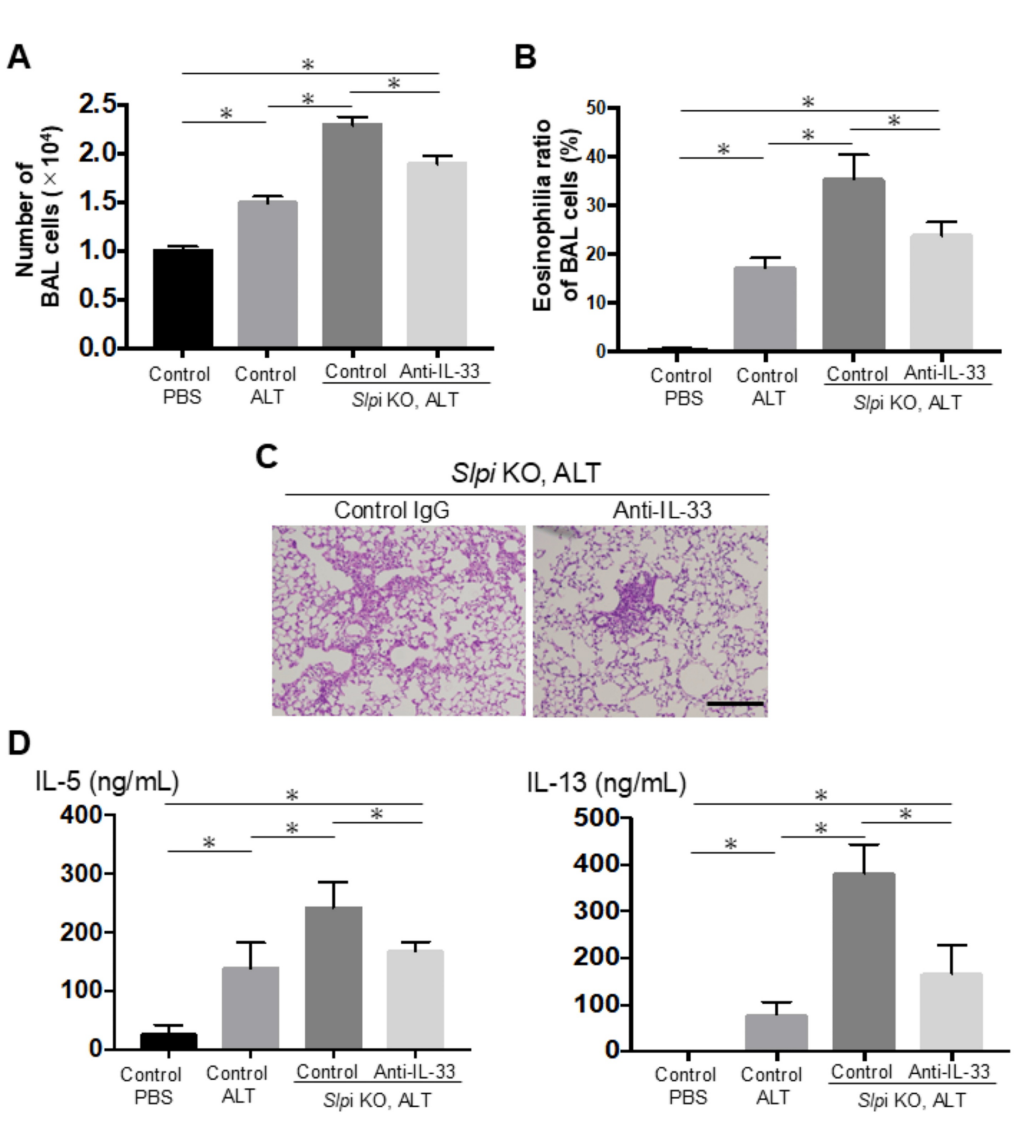
IL-33 plays a key role in the augmented type 2 airway inflammation caused by SLPI downregulation. WT and *SLPI* KO mice were intranasally administered anti-IL-33 antibody or control IgG following *Alternaria *extract or PBS treatment on days 1-3. All analyses were performed on day 4 (n = 3). (A, B) Airway inflammation was evaluated by the percentage of eosinophils in BAL fluid. (C) Representative lung histology sections stained with H&E (scale bar: 200 μm). (D) Cytokine levels (IL-5 and IL-13) in BAL fluid. Data are shown as mean ± SEM. Unpaired Student’s t-test (for two groups) and Tukey’s post hoc test (for multiple comparisons); (asterisks indicate p < 0.05).

In addition, we used an in vivo mouse model with therapeutically administered anti-IL-33 neutralizing antibody to determine whether IL-33 plays a key role in the augmented Th2 airway inflammation caused by downregulation of *SLPI* expression. We intranasally treated mice with anti-IL-33 neutralizing antibody 1 hour after an *Alternaria* extract injection to SLPI KO mice because IL-33 peaks in the airway 1 hour after an allergen challenge. In contrast to mice treated with the control IgG antibody to *SLPI* KO mice, *SLPI* KO mice treated with anti-IL-33 neutralizing antibody showed reduced airway inflammation and fewer numbers of BAL total cells, ratio of eosinophilia, and Th2 cytokines (e.g., IL-5 and IL-13) in BAL fluid (Figures 4A-4D).

Inhibition of serine protease attenuated the Th2 airway inflammation after *Alternaria* exposure under the downregulation of SLPI expression

**Figure 5:**
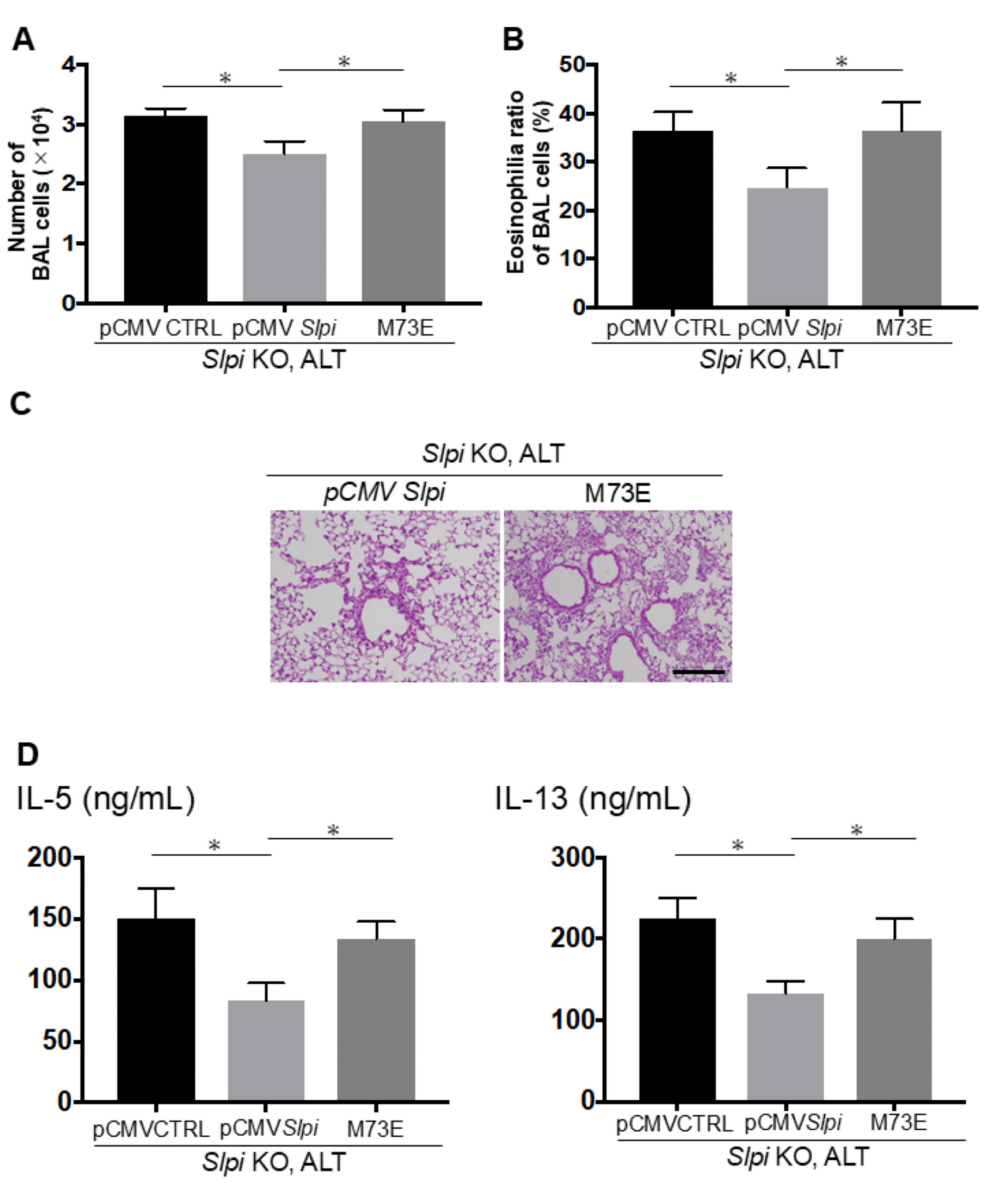
SLPI, as a serine protease inhibitor, attenuates Th2 airway inflammation following Alternaria exposure. These experiments were performed using a similar protocol to that of Figure 1. *SLPI* KO mice were intranasally transfected with 50 μg of a plasmid vector (pmCMV) encoding wild-type *SLPI*, mutant *SLPI* (M73E), or empty control (n = 3). (A, B) Airway inflammation was assessed via total BAL cell counts (A) and eosinophil ratios (B). (C) Representative lung histology sections stained with H&E (scale bar: 200 μm). (D) IL-5 and IL-13 cytokine levels in BAL fluid are measured by ELISA. Data are shown as mean ± SEM. Unpaired Student’s t-test (for two groups) and Tukey’s post hoc test (for multiple comparisons); (asterisks indicate p < 0.05).

**Figure 6:**
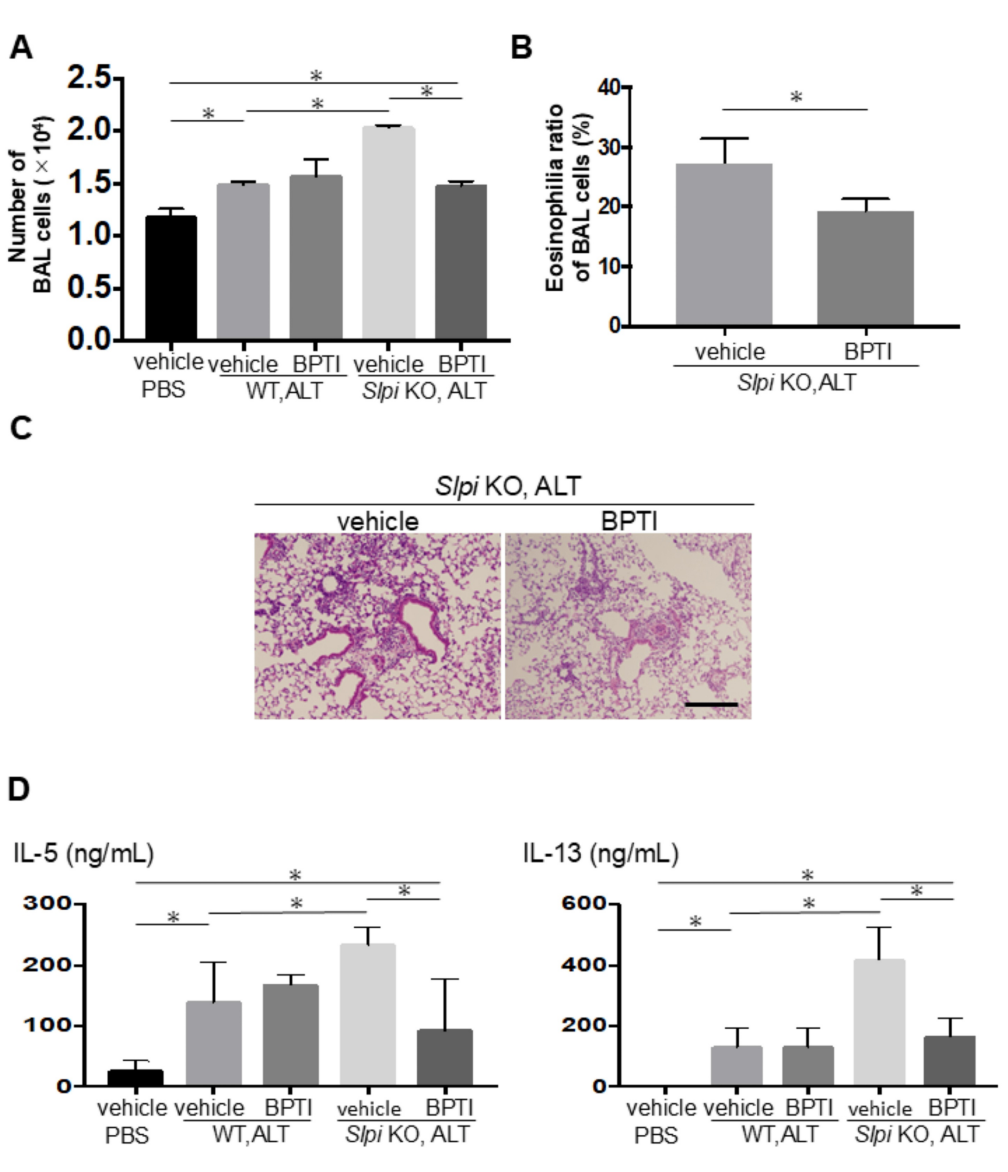
Inhibition of serine protease attenuates Th2 airway inflammation with reduced SLPI expression following Alternaria extract exposure. WT and *SLPI* KO mice were intranasally administered BPTI or vehicle following the presence or absence of Alternaria extract (or PBS) treatment on days 1-3. All analyses were conducted on day 4 (n = 3). (A, B) Airway inflammation was evaluated by total BAL cell counts (A) and eosinophil percentage (B; only SLPI KO shown). (C) Representative lung sections stained with hematoxylin and eosin (H&E; scale bar: 200 μm). (D) IL-5 and IL-13 cytokine levels in BAL fluid measured by ELISA. Data are shown as mean ± SEM. Unpaired Student’s t-test (for two groups) and Tukey’s post hoc test (for multiple comparisons); (asterisks indicate p<0.05).

Recent reports suggest that *Alternaria* extract-induced serine protease promotes IL-33 activation, exacerbating Th2 airway inflammation [6]. To investigate whether SLPI’s function as a serine protease inhibitor is crucial for the augmented Th2 airway inflammation caused by downregulation of *SLPI* expression, we constructed a single amino acid substitution mutant of mouse *SLPI* (M73E: Met73Gln). In this mutant, the plasmid lacks functional anti-protease activity because the protease-inhibiting region of *SLPI* is localized between residues 67-74 in the WAP II domain (C-terminal), and we transfected it into *SLPI* KO mice. After injection with pCMV SLPI (wild-type SLPI), *Alternaria*-treated SLPI KO mice displayed airway inflammation evaluated by histological examination, total BAL cell count, the ratio of eosinophilia in BAL, and levels of Th2 cytokines including IL-5 and IL-13 in BAL fluid, which were significantly decreased compared to those injected with pCMV CTRL (Figures 5A-5D). In contrast, *SLPI* KO mice transfected with the M73E mutant did not show these protective effects, indicating that SLPI’s protease-inhibitory activity is required for suppressing airway inflammation (Figures 5A-5D). To confirm the key function of SLPI as a serine protease inhibitor for *Alternaria* extract-induced airway inflammation in *SLPI* KO mice, we next investigated the effects of BPTI, a serine protease inhibitor, in this model. Mice received intranasal BPTI 1 hour after each *Alternaria* injection for 3 days. In contrast to *SLPI* KO mice treated with the control solvent, BPTI-treated *SLPI* KO mice showed reduced airway inflammation, fewer total BAL cells, and lower ratios of eosinophilia and Th2 cytokines, including IL-5 and IL-13, in BAL fluid (Figures 6A-6D).

Decreased SLPI expression increased the release and cleavage of IL-33 after *Alternaria* exposure in human bronchial epithelial cells

**Figure 7:**
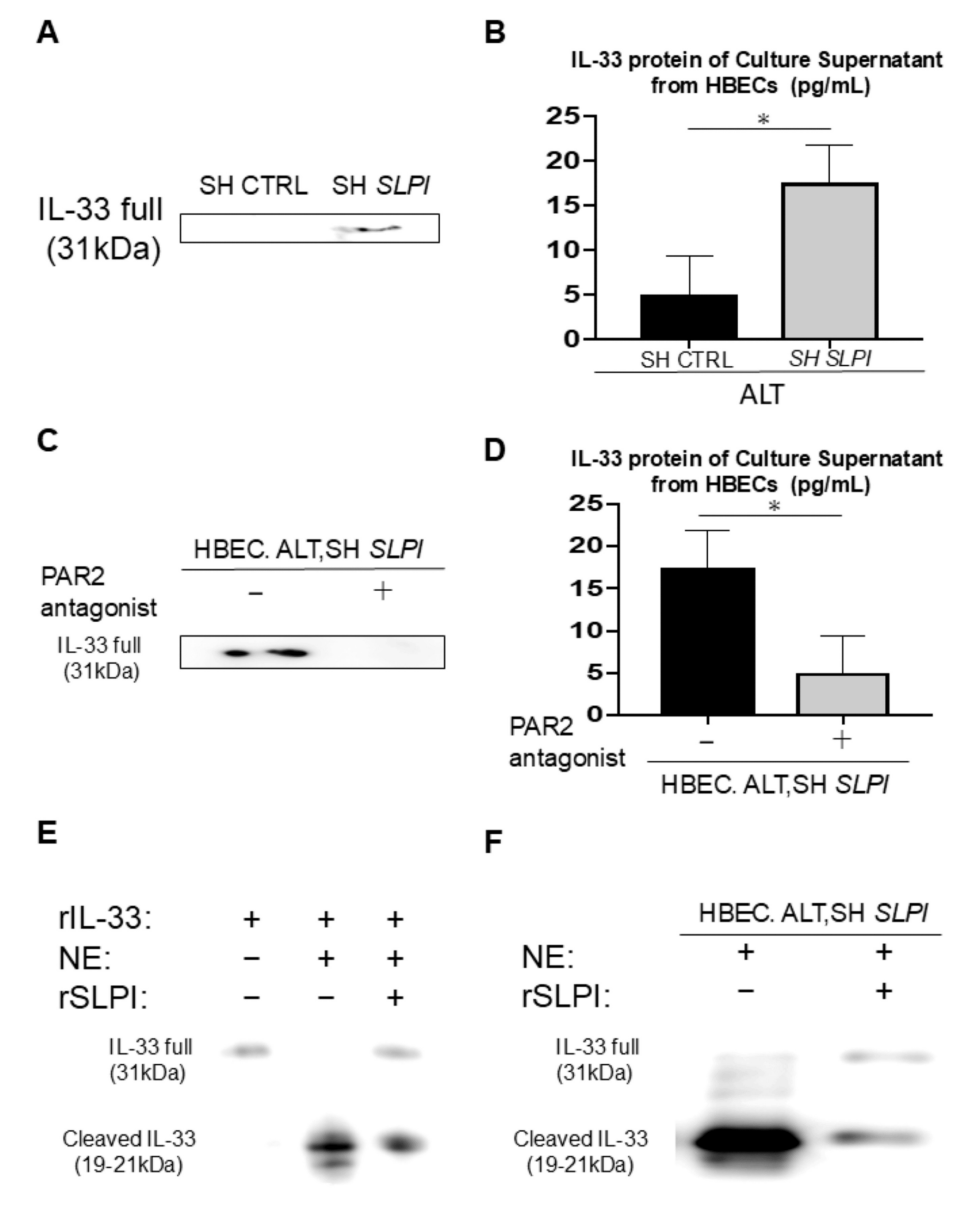
Decreased SLPI expression enhances IL-33 release and cleavage in human bronchial epithelial cells following Alternaria exposure. HBECs were transfected with shSLPI or CTRL followed by exposure to *Alternaria* extract (100 µg/mL) for 4 h. (A) Western blot of IL-33 in culture supernatants. (B) ELISA quantification of IL-33 in culture supernatants (n = 3). (C, D) HBECs transfected with sh*SLPI* were treated with or without a PAR-2 antagonist following *Alternaria* exposure. (C) Western blot of IL-33 in culture supernatants. (D) ELISA quantification of IL-33 in culture supernatants (n = 3). (E) Recombinant IL-33 (1 µg) was incubated with neutrophil elastase (NE, 0.3 U, 30 min at 37°C) with or without recombinant SLPI (100 µg), and Western blot was performed. (F) HBECs in vitro *SLPI* knockdown model with shRNA were treated with NE (0.3 U, 30 min, 37°C) with or without recombinant SLPI following *Alternaria* exposure, and Western blot was perfomed. Data are shown as mean ± SEM. Unpaired Student’s t-test for two groups; asterisks indicate p<0.05.

To clarify whether human SLPI is also involved in the release of IL-33, we next used an in vitro model of *Alternaria* extract exposure to HBECs from normal subjects. We then transfected HBECs with plasmid vectors expressing psh*SLPI* or CTRL. Western blot and ELISA analyses showed that *Alternaria* extract-induced IL-33 protein from the culture supernatant was increased after *SLPI*-knockdown HBECs compared to those transfected with pshCTRL (Figures 7A, 7B). A recent report showed that serine proteases derived from *Alternaria* extract promote the release of IL-33 through activation of PAR-2. We then evaluated the effect of PAR-2 in this model. Western blot and ELISA analyses showed that treatment with a PAR-2 antagonist significantly reduced *Alternaria* extract-induced IL-33 protein release in *SLPI*-knockdown HBECs compared to vehicle control (Figures 7C, 7D). Next, we investigated whether human recombinant SLPI suppresses the human NE-mediated cleavage of recombinant IL-33FL, which increases the IL-33 activity. Western blot analyses revealed that NE treatment cleaved full-length recombinant human IL-33 (31 kDa) into shorter mature forms of 18-21 kDa. In contrast, co-treatment with recombinant SLPI partially prevented NE-induced cleavage of IL-33FL to shorter mature forms (Figure 7E). To determine the functional consequences of SLPI suppressing the NE-mediated cleavage of recombinant IL-33FL in this model, we finally investigated whether human recombinant SLPI also suppresses the cleavage of the *Alternaria* extract-induced release of IL-33FL from the HBECs in vitro *SLPI* knockdown model with shRNA by human NE. A similar trend was observed, as in Figure 7E. Western blot analysis revealed that NE-mediated cleaved IL-33FL from the HBECs in vitro *SLPI* knockdown model with shRNA (31 kDa), into shorter mature forms. In contrast, co-treatment with recombinant SLPI partially prevented NE-induced cleavage of IL-33FL from the HBECs in vitro SLPI knockdown model with shRNA to shorter mature forms (Figure 7F).

## Discussion

Our goal in this study was to determine whether SLPI contributes to the protective regulation of protease-dependent IL-33-induced type 2 immunity through an innate immune response in vivo. In this context, we demonstrated that *SLPI* downregulation (in *SLPI* KO and shRNA knockdown models) potentiates *Alternaria* extract-induced Th2 airway inflammation in an IL-33-dependent manner. We also demonstrated that exposure to *Alternaria* extract in *SLPI* KO mice increased the percentage and number of ILC2s. These findings underscore the importance of SLPI’s serine protease inhibitor function in regulating Th2 airway inflammation following *Alternaria* extract exposure. In *SLPI* KO mice, transfection with wild-type *SLPI* (pmCMV-*SLPI*), but not a single amino acid mutant (M73E: Met73Gln), which lacks protease-inhibitory activity, significantly suppressed inflammation, and administration of BPTI, a serine protease inhibitor, also attenuated *Alternaria*-induced Th2 airway inflammation in SLPI KO mice, thereby supporting the therapeutic potential of targeting protease activity. Our in vitro model using HBECs demonstrated that SLPI functions as a serine protease inhibitor protectively by inhibiting excessive IL-33FL release and cleavage into shorter mature forms. Collectively, these findings demonstrate that reduced SLPI levels enhance *Alternaria* extract-induced Th2-type airway inflammation depends on both IL-33FL release and cleavage into shorter mature forms, and it is mediated by expansion of activated ILC2s.

In asthma patients, SLPI levels in nasal lavages were significantly higher than those in the controls, as has been reported [17]. However, the mRNA of SLPI in the airway epithelial cells in patients with severe asthma was significantly lower compared to those with mild or moderate asthma [12]. Furthermore, scientific reports using ovalbumin or IgE, which is a Th2-type inflammation mouse model, demonstrated that downregulation of the SLPI gene contributed to increasing Th2-type inflammation [10,11]. These reports suggested that SLPI in asthma patients may exhibit an anti-inflammatory function in mild to moderate asthma, while a low SLPI level may contribute to exacerbating the Th2-type airway inflammation in severe asthma patients [12]. Consistent with these findings, our results further support that a low level of SLPI potentiates Th2 airway inflammation via the IL-33-dependent pathway through an innate immune response in models of airway inflammation. 

Recent studies have shown that IL-33 plays a central role in promoting Th2 airway inflammation by activating type 2 immunity in asthma patients. Concerning how reduced SLPI levels might contribute to IL-33 production, an in vitro study showed the inhibitory effect of SLPI on ATP-induced IL-33 production in type 2 alveolar epithelial cells [13]. However, whether this mechanism also operates in vivo remains unclear. Recently, emerging evidence concerning the relationship between type 2 innate immunity and serine protease indicates that protease activity possessed by *Alternaria* extract promotes the exacerbation of TH2-type airway inflammation [6,18]. SLPI acts as a serine protease inhibitor on NE, and it protects airway inflammation from excessive protease activity in vivo [8,9,19]. We therefore focused on SLPI as a key regulator of type 2 immunity in an IL-33-dependent manner. As expected, in this context, our findings demonstrate that SLPI, acting as a serine protease inhibitor, not only contributes to the type 2 innate immune response in vivo but also represents a potential therapeutic target for conditions with reduced SLPI expression.

Our two in vitro models (using recombinant IL-33 and IL-33 from *Alternaria* extract-treated HBECs) demonstrated that SLPI is involved in both IL-33 release from HBECs and NE-mediated cleavage of IL-33FL to shorter forms, which increases the IL-33 activity. Recent evidence has shown that *Alternaria* extract-induced serine protease promotes both the release and cleavage of IL-33FL to shorter mature forms. Concerning the regulation of the release of IL-33 by serine protease, previous reports have shown that epithelial tissue damage caused by *Alternaria* extract-induced serine protease leads to enhanced IL-33 release via the PAR-2 pathway [16,20]. We therefore evaluated the suppressive regulation of the release of IL-33 by PAR-2 antagonist under low SLPI level. Our in vitro model of *Alternaria* extract exposure to HBECs under reduced SLPI level showed that PAR-2 antagonist reduced the release of IL-33 compared to the control solvent. Our results and others suggest that the serine protease imbalance caused by reduced SLPI level activates the PAR-2 pathway, which may augment the release of IL-33 from epithelial cells. Concerning the regulation of IL-33FL cleavage, multiple allergens, including *Alternaria alternata*, have been shown to possess protease activity and directly or indirectly cleave IL-33FL [16]. We therefore evaluated whether SLPI, as a serine protease inhibitor, suppresses protease activity and thereby protects against the IL-33FL cleavage. As expected, our two in vitro models showed that SLPI also showed protective regulation of the cleavage of IL-33FL to suppress protease activity.

This study has several limitations that should be acknowledged. First, while we focused on the impact of low levels of SLPI on the IL-33 pathway, other important inflammatory pathways were not thoroughly evaluated. For instance, alarmins such as IL-25 and thymic stromal lymphopoietin, which are also secreted by airway epithelial cells and contribute to type 2 inflammation similarly to IL-33, were not assessed in the current study. Second, the assessment of SLPI-mediated protection against IL-33FL cleavage was conducted only in vitro. We did not evaluate endogenous IL-33 protein degradation kinetics in vivo in BAL fluid. Lastly, the administration of BPTI, a serine protease inhibitor, to WT mice did not produce a therapeutic effect on *Alternaria* extract-induced Th2 airway inflammation compared to vehicle control. We speculate that this lack of therapeutic effect may be due to sufficient levels of endogenous SLPI in WT mice, which already provides effective inhibition of serine protease activity.

## Conclusions

Collectively, although we demonstrated the possibility of a novel mechanism by which SLPI suppresses protease-dependent IL-33 activation and regulates the expansion of ILC2s under low SLPI levels based on findings from two *SLPI* gene downregulation models (*SLPI* KO mice and an in vivo *SLPI* knockdown model with shRNA) and two intervention models (treatment with an anti-IL-33 neutralizing antibody or the BPTI), further studies are needed to evaluate its regulatory effects in human systems and downstream cytokine pathways in vivo. These results may provide a foundation for developing novel therapeutic approaches targeting protease regulation in type 2 innate immunity through the control of serine protease imbalance in asthma patients with reduced SLPI expression.

## References

[1] Brusselle GG, Koppelman GH (2022). Biologic therapies for severe asthma. N Engl J Med.

[2] Préfontaine D, Nadigel J, Chouiali F (2010). Increased IL-33 expression by epithelial cells in bronchial asthma. J Allergy Clin Immunol.

[3] Halim TY, Steer CA, Mathä L (2014). Group 2 innate lymphoid cells are critical for the initiation of adaptive T helper 2 cell-mediated allergic lung inflammation. Immunity.

[4] Moffatt MF, Gut IG, Demenais F (2010). A large-scale, consortium-based genomewide association study of asthma. N Engl J Med.

[5] Molofsky AB, Savage AK, Locksley RM (2015). Interleukin-33 in Tissue Homeostasis, Injury, and Inflammation. Immunity.

[6] Snelgrove RJ, Gregory LG, Peiró T (2014). Alternaria-derived serine protease activity drives IL-33-mediated asthma exacerbations. J Allergy Clin Immunol.

[7] Cayrol C, Duval A, Schmitt P (2018). Environmental allergens induce allergic inflammation through proteolytic maturation of IL-33. Nat Immunol.

[8] Nakamura A, Mori Y, Hagiwara K (2003). Increased susceptibility to LPS-induced endotoxin shock in secretory leukoprotease inhibitor (SLPI)-deficient mice. J Exp Med.

[9] Nukiwa T, Suzuki T, Fukuhara T, Kikuchi T (2008). Secretory leukocyte peptidase inhibitor and lung cancer. Cancer Sci.

[10] Marino R, Thuraisingam T, Camateros P (2011). Secretory leukocyte protease inhibitor plays an important role in the regulation of allergic asthma in mice. J Immunol.

[11] Matsuba S, Yabe-Wada T, Takeda K (2017). Identification of secretory leukoprotease inhibitor as an endogenous negative regulator in allergic effector cells. Front Immunol.

[12] Raundhal M, Morse C, Khare A (2015). High IFN-γ and low SLPI mark severe asthma in mice and humans. J Clin Invest.

[13] Draijer C, Hylkema MN, Boorsma CE (2016). Sexual maturation protects against development of lung inflammation through estrogen. Am J Physiol Lung Cell Mol Physiol.

[14] Santoso A, Kikuchi T, Tode N (2016). Syndecan 4 mediates Nrf2-dependent expansion of bronchiolar progenitors that protect against lung inflammation. Mol Ther.

[15] Hirano T, Kikuchi T, Tode N (2016). OX40 ligand newly expressed on bronchiolar progenitors mediates influenza infection and further exacerbates pneumonia. EMBO Mol. Med.

[16] Shishikura Y, Koarai A, Aizawa H (2016). Extracellular ATP is involved in dsRNA-induced MUC5AC production via P2Y2R in human airway epithelium. Respir Res.

[17] Belkowski SM, Boot JD, Mascelli MA (2009). Cleaved secretory leucocyte protease inhibitor as a biomarker of chymase activity in allergic airway disease. Clin Exp Allergy.

[18] Boitano S, Flynn AN, Sherwood CL (2011). Alternaria alternata serine proteases induce lung inflammation and airway epithelial cell activation via PAR2. Am J Physiol Lung Cell Mol Physiol.

[19] Jaeger N, McDonough RT, Rosen AL (2022). Airway microbiota-host interactions regulate secretory leukocyte protease inhibitor levels and influence allergic airway inflammation. Cell Rep.

[20] Nishimura R, Miyajima M, Takahashi K (2022). House dust mite-derived serine protease upregulates gene expression of interleukin-33 in canine keratinocytes via protease-activated receptor-2. Vet Dermatol.

